# Changes in lipid composition during sexual development of the malaria parasite *Plasmodium falciparum*

**DOI:** 10.1186/s12936-016-1130-z

**Published:** 2016-02-06

**Authors:** Phuong N. Tran, Simon H. J. Brown, Melanie Rug, Melanie C. Ridgway, Todd W. Mitchell, Alexander G. Maier

**Affiliations:** Research School of Biology, The Australian National University, Canberra, ACT Australia; La Trobe Institute of Molecular Science, La Trobe University, Melbourne, VIC Australia; School of Medicine and Illawarra Health and Medical Research Institute, University of Wollongong, Wollongong, NSW Australia; Centre for Advanced Microscopy, The Australian National University, Canberra, ACT Australia

**Keywords:** Malaria, *Plasmodium falciparum*, Lipids, Gametocytes, Host cell remodelling

## Abstract

**Background:**

The development of differentiated sexual stages (gametocytes) within human red blood cells is essential for the propagation of the malaria parasite, since only mature gametocytes will survive in the mosquito’s midgut. Hence gametocytogenesis is a pre-requisite for transmission of the disease. Physiological changes involved in sexual differentiation are still enigmatic. In particular the lipid metabolism—despite being central to cellular regulation and development—is not well explored.

**Methods:**

Here the lipid profiles of red blood cells infected with the five different sexual stages of *Plasmodium falciparum* were analysed by mass spectrometry and compared to those from uninfected and asexual trophozoite infected erythrocytes.

**Results:**

Fundamental differences between erythrocytes infected with the different parasite stages were revealed. In mature gametocytes many lipids that decrease in the trophozoite and early gametocyte infected red blood cells are regained. In particular, regulators of membrane fluidity, cholesterol and sphingomyelin, increased significantly during gametocyte maturation. Neutral lipids (serving mainly as caloriometric reserves) increased from 3 % of total lipids in uninfected to 27 % in stage V gametocyte infected red blood cells. The major membrane lipid class (phospholipids) decreased during gametocyte development.

**Conclusions:**

The lipid profiles of infected erythrocytes are characteristic for the particular parasite life cycle and maturity stages of gametocytes. The obtained lipid profiles are crucial in revealing the lipid metabolism of malaria parasites and identifying targets to interfere with this deadly disease.

**Electronic supplementary material:**

The online version of this article (doi:10.1186/s12936-016-1130-z) contains supplementary material, which is available to authorized users.

## Background

Intraerythrocytic sexual stages of human malaria parasites are essential for the transmission of *Plasmodium falciparum* from human host to mosquito. In contrast to the 48-hour blood stage life cycle of asexual parasites, it takes 9–12 days for *P. falciparum* gametocytes to fully develop inside human red blood cells (RBCs). During this time the gametocytes progress through five morphologically distinct stages. The prolonged duration of gametocyte maturation is a unique feature of only a few *Plasmodium* species infecting higher primates (*P. falciparum* and *P. reichenowi*) [1, 2]. Infection with *P. falciparum* causes by far the highest morbidity of all human *Plasmodium* species. During the intraerythrocytic development, asexual parasites modify the host cell membrane extensively by exporting a range of proteins [3]. These modifications include a marked increase in adhesiveness [3] and changes in fluidity [4–6], fatty acid composition [7–10], phospholipid organization [11–13] and permeability [14–17] of the parasitized host cell membrane. In contrast, little is known about host cell remodelling by gametocytes. In recent studies, the membranes of RBCs infected with later stage gametocytes (stage IV and V) have been found to be more deformable than those of the disease causing asexual parasites [18–20].

Membrane lipids of RBCs are believed to be the foundation of these cells’ exquisite stability and deformability [21]. The biconcave disk shaped human cells are devoid of a nucleus and of most cellular organelles. Upon infection and development of the asexual parasite the surface of the RBC membrane displays membrane protrusions—so called knobs, which harbour the virulence factor PfEMP1 that mediates cytoadhesion. The volume of the RBC increases only slightly (by ~17 %) while the parasite develops into the trophozoite stage [22]. However, at the same time the parasite occupation within the RBC volume changes from initially 4 to 80 % [23]. There is enormous demand for lipids due to parasite growth inside the host cell [developing organelles (e.g. nucleus, mitochondria, food vacuoles and apicoplast) surrounded by membranes and accumulating lipids in lipid bodies] and subsequent replication. Additionally, new lipid structures also appear inside the host cell: the parasite is surrounded by a parasitophorous vacuole membrane [24] and new parasite induced membranous structures (Maurer’s clefts and transport vesicles) emerge, which play an important role in trafficking of parasite virulence factors to the surface of infected red blood cell (iRBC) membranes.

Upon induction of gametocytes the infected red blood cell undergoes remarkable modifications: initially the cell morphology is indistinguishable from asexual infected RBC, but in later stages the gametocyte-infected RBC elongate before rounding up again. Especially in the more mature stages most of the host cell volume is taken up by the parasite. Inside the parasite lipid accumulations, in the form of lipid bodies and osmiophilic bodies, can be observed. The latter are more pronounced in the female form and are named for their intensive lipid staining by osmium tetroxide.

Most of the observed increases in lipids in *Plasmodium*-infected red blood cells can be attributed to plasmodial membrane phospholipids. Despite the high lipid demand for extensive proliferation of the asexual parasites, the parasite cannot de novo synthesize cholesterol [7, 10, 25, 26] and has only limited capacity for fatty acid synthesis [27]. Furthermore, the host RBCs themselves are also incapable of de novo biosynthesizing fatty acids [28], cholesterol or phospholipids [29]. Therefore, the parasites actively scavenge these lipid precursors from the serum to metabolize them into the required lipids [30, 31]. In *P. falciparum*, host-derived cholesterol and parasite-driven biosynthesis of sphingolipids and phospholipids are crucial for intraerythrocytic development [32–35]. Hence, molecules involved in biosynthesis of lipids were suggested to be attractive targets for novel chemotherapies against *P. falciparum* [34, 36–38].

In contrast to the considerable number of reports on the lipid composition and metabolism in the asexual blood-stages of the malaria parasite, essentially nothing is known about the lipid composition of the sexual stages and its alterations during sexual development. Bobenchik et al. recently supplied genetic and pharmacological evidence that the synthesis pathway for phosphatidylcholine is essential for gametocytogenesis and transmission to mosquitoes and novel anti-malarial compounds have been identified targeting enzymes involved in the lipid synthesis [39, 40].

In this study, it was hypothesized that the profound and extraordinary morphological changes seen during gametocyte maturation is reflected in the lipid composition. The lipid profiles of RBCs infected with the five different sexual stages of *P. falciparum,* therefore, were comprehensively analysed by mass spectrometry and compared to those obtained from uninfected and asexual trophozoite infected RBCs. Understanding the biological concepts behind the contribution of lipids to the sexual parasite growth will allow the development of novel approaches to prevent malaria transmission. Comparing the lipid composition of related apicomplexan parasites and their different hosts and vectors might highlight crucial lipid species, possibly responsible for the host and vector specificity of *Plasmodium.*

## Methods

### Parasite culture

Parasites of the *P. falciparum* 3D7 strain were cultured using the standard methods with slight modifications [41, 42]. Parasites were maintained in O+ RBCs at 4 % haematocrit resuspended in RPMI medium supplemented with 10 mM glucose, 480 μM hypoxanthine, 20 μg/mL gentamycin and 10 % (v/v) human serum. Cultures were maintained at 37 °C under microaerophilic conditions (1 % O_2_, 5 % CO_2_, 94 % N_2_). Asexual parasites were synchronized by a double sorbitol treatment [43]. Fresh frozen plasma was pooled from at least five different donors and heat-inactivated.

Uninfected RBCs were collected in CPD buffer (26.3 g/L sodium citrate dihydrate, 3.27 g/L citric acid monohydrate, 2.51 g/L monobasic sodium phosphate dihydrate, 25.5 g/L dextrose monohydrate) and used within 7 days of collection. RBCs for analysis were incubated in culture media and the same conditions as parasite infected RBCs.

### Gametocyte commitment and harvesting

Induction of gametocytogenesis and collection of highly synchronous gametocytes were performed as described previously [44] with slight modifications. Briefly, 2 % synchronous trophozoites were incubated overnight to obtain about 8–10 % ring stage parasites. In order to induce sexual development, the parasites were fed with medium containing 75 % of the spent parasite-conditioned medium. On the next day (designated as day-1) when parasites reached trophozoite stage, the culture was split to obtain 2 % trophozoites with the retention of one-third of the spent medium. After overnight incubation, about 8–10 % “stressed” ring-stage parasites appeared in the culture the next day, which was designated as day 0. All mature asexual parasites were excluded by magnet purification (CS and D columns; MACS Cell Separation, Miltenyi Biotec), while all ring-stage parasites containing both sexual and asexual ring stages were collected in the flow-through and then split to 2 % parasitaemia.

Subsequently, the medium was replaced daily with fresh medium containing 50 mM N-Acetyl-D-glucosamine (GluNAc) to kill all mature asexual parasites [45]. In order to restrict the growth of the asexual parasites during the early period of GluNAc treatment, the culture was treated with 5 % sorbitol on the following day (day +1) to eliminate all the mature asexual parasites, but not the committed gametocytes [46]. The culture was maintained for 12 days until gametocytes became mature. Within one biological replicate samples from the same culture were collected; three independent biological replicates were analysed. Stage I gametocytes were collected on day 2 of commitment, with additional stages collected every 2 days thereafter. Gametocytes were magnet purified with gametocyte infected RBC sticking to the magnet column. All dead cells and uRBC were washed out with pre-warmed media. Gametocytes were then eluted. Purity (>95 %) and stage were checked by Giemsa-stained thin smears and Western blotting using the gametocyte marker Pfs16 [see Additional file 1].

### Lipid labelling assay

Cellular cholesterol was labelled by washing cells three times in PBS followed by an incubation in PBS containing 5 μM 25-NBD-cholesterol (Avanti Polar) (10 mM stock made in 100 % ethanol) at 37 °C for 1 h. For the neutral lipid labelling cells were washed three times with PBS before being fixed with 4 % formaldehyde in PBS at RT for 20 min. The cells were then washed three times with PBS and incubated with PBS containing 1 × LipidTOX Red Neutral Lipid Stain (Invitrogen) at RT for 30 min.

Polar lipids were labelled washing the cells three times in PBS and incubating them in a 1 μg/mL Nile Red (Sigma) solution in PBS (1 mg/ml stock in 100 % acetone) at 37 °C for 1 h.

With the exception of the cholesterol labelled cells, which were imaged with a filter set for Alex Fluor 488 dye (green), all labelled cells were imaged with a filter set for Alex Fluor 594 dye (red) on a deconvolution microscope (DeltaVision Elite, Applied Precision). All images were recorded at the same setting and exposure time. Images were deconvolved using the softWoRx acquisition software (version 5.0) and then processed with ImageJ 1.43u software (NIH, USA).

### Lipidomic analysis

Synchronous parasitized RBCs were enriched using magnetic cell separating columns (CS and D columns; MACS Cell Separation, Miltenyi Biotec), and then counted on a haemocytometer. Lipid extraction was performed according to the method of Matyash et al. [47]. Analysis was performed on three independent cell harvests (serving as biological replicates).

In brief, approximately 10^7^ of magnet-purified cells were added to 2 mL tough tubes (Geneworks, Hindmarsh, SA, Australia), fixed with 300 μL of methanol and stored at −80 °C until extraction step. An aliquot of 100 μL of methanol containing 0.01 % butylated hydroxytoluene and internal standards (see Additional file 2) were added to each tube. Samples were homogenized using a bead homogenizer (FastPrep-24, MP Biomedical, Seven Hills, NSW, Australia) at 6 m/s for 40 s and the homogenate was transferred to 2 mL microcentrifuge tubes (Eppendorf, North Ryde, NSW, Australia). Beads were washed with 100 μL of methanol and the wash added to the homogenate. One millilitre of methyl-tert butyl ether was added and samples were vortexed at 4 °C for 1 h. To induce phase separation 300 μL of 150 mM ammonium acetate (LC–MS grade, Fluka, Castle Hill, NSW, Australia) was added. Tubes were vortexed for 15 min and spun at 2000*g* for 5 min to complete phase separation. Eight hundred μL of the upper organic layer was transferred to a new 2 mL glass vial and stored at −20 °C until analysis. Extracts were diluted 40-fold into methanol:chloroform (2:1 v/v) containing 5 mM ammonium acetate prior to mass spectrometric analysis.

Mass spectra were acquired using a chip based nano-electrospray ionization source (TriVersa Nanomate^®^, Advion, Ithaca, NY, USA) coupled to a hybrid linear ion trap- triple quadrupole mass spectrometer (QTRAP^®^ 5500, ABSCIEX, Foster City, CA, USA) [48]. Ten microliter of extract was aspirated from a sealed 96-well plate (Eppendorf Twin- Tec) and delivered into the mass spectrometer via a nano-electrospray ionization (ESI) chip with an orifice diameter of 4.1 μm. The delivery gas was nitrogen at a pressure of 0.4 psi and a spray voltage of 1.15 kV was used for positive ion acquisition. Target lipids and MS scan parameters are shown in Additional file 3. Experimental conditions for positive ion mode acquisition were a declustering potential of 100 V, entrance potential of 10 V and a scan rate of 200 *m/z* units.s^-1^. Mass spectra were averaged over 50 scans.

Data were analysed with LipidView^®^ (ABSCIEX) software version 1.2, including smoothing, identification, removal of isotope contribution from lower mass species, and correction for isotope distribution. Ionized lipids detected with a signal-to-noise ratio (s/n) over 20 were included in the analysis. Extraction and solvent blanks were included in the analysis to allow exclusion of ions detected at lipid masses that result from extraction chemical or solvent impurities. Quantification was achieved in LipidView^®^ software by comparison of the peak area of individual lipids to their class-specific internal standards after isotope correction. Ion detected at masses that could be assigned to odd-chain fatty acid, phospholipids or ether-linked phospholipids were assumed to be ether-linked. Ether-linked PE species produce the 141 head group fragments at approximately 29 % of the efficiency of di-acyl PE species [49]; therefore, a correction factor of 3.45 was applied to all ether-PE species [50]. Sphingomyelin species were detected using the 184.1 *m/z* precursor ion scan, which cannot distinguish between isobaric DHSM and SM species. Ion detected at masses that could be assigned to DHSM or SM were assumed to contain the major SM backbone d18:1, and were listed as species indicating the respective N-linked fatty acid. Lipid molecular species were notated in accordance with the recently proposed shorthand by Liebisch et al. [51], except for DAG and TAG.

### Statistics

Data were presented as mean ± standard deviation (SD). To determine differences between seven groups of cells based on their total lipid, phospholipid, sphingolipid, cholesterol or neutral lipid levels, one-way ANOVA test or unpaired Student’s *t* test was performed using GraphPad Prism 5.0. Principal component analysis (PCA) data processing was performed using MarkerView v1.2.1.1 (Applied Biosystems | MDS Sciex, Toronto, Ontario, Canada). Data from LipidView were exported to Microsoft^®^ Excel (v14.3.9), group labels were added, and data were imported into Markerview with lipid ID and quantification included. Data were scaled using Pareto scaling and no weighting was applied. PCA was performed in an unsupervised mode. Data were graphed using GraphPad Prism 5.0 and Microsoft^®^ Excel (v14.3.9).

## Results

### Distinct morphological lipid pattern of *P. falciparum* gametocytes

**Fig. 1:**
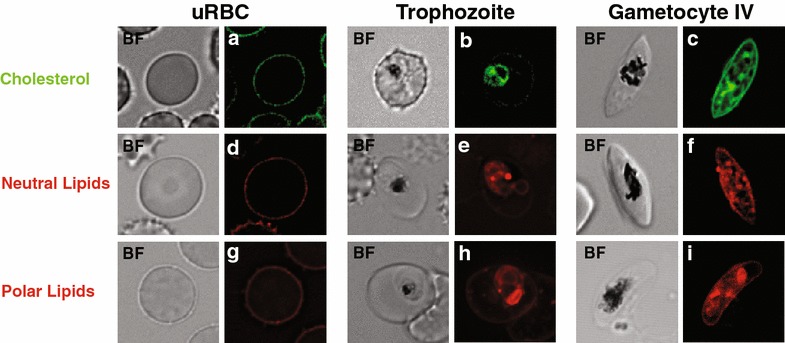
Blood-stage *P. falciparum* parasites show different lipid pattern. Lipid fluorescent labelling of the blood-stage parasites. Fluorescence imaging of uninfected RBCs (**a**, **d**, **g**), trophozoite infected RBCs (**b**, **e**, **h**) and gametocyte infected RBCs (**c**, **f**, **i**) stained with 25-NBD cholesterol (to label cholesterol), LipidTOX Red (to label neutral lipids) and Nile Red (to label polar lipids) was performed on a DeltaVision Elite microscope (Applied Precision) at the same fluorescent recording setting. *uRBC* uninfected red blood cells, *BF* bright field.* Scale bar*: 5 μm

In order to visualize and distinguish the overall changes in the lipid pattern of RBCs infected with gametocytes compared to uninfected and trophozoite-infected RBCs, the cells were labelled with different lipid probes for cholesterol, neutral lipids and polar lipids and imaged at the same settings. In uninfected RBCs all forms of lipids are predominantly present in the RBC plasma membrane (Fig. 1).

The plasma membrane of uninfected human RBCs is especially rich in free cholesterol (Fig. 1a) [26]. Cholesterol modulates the membrane fluidity and reduces the permeability of the plasma membrane to solutes like hydrogen and sodium ions [52]. In contrast upon infection only a very faint signal was detected in the plasma membrane of RBCs infected with trophozoite-stage parasites when stained with 25-NBD cholesterol [53] (Fig. 1b). Accumulation of cholesterol within the parasite was reminiscent of lipid bodies, in addition to the immediate surrounding of the parasite [parasite plasma membrane and/or the parasitophorous vacuole membrane (PVM)] (Fig. 1b, c). This is consistent with previous reports, which located cholesterol inside blood-stage *P. falciparum* cells primarily in lipid bodies and rhoptries [54, 55], [56–58].

Neutral lipid staining with LipidTOXRed (Life Technologies) showed only weak labelling of the plasma membrane in uninfected RBCs (Fig. 1d), but revealed brightly labelled round structures apparent in the parasite’s cytosol upon maturation of asexual and sexual parasites. These structures likely represent lipid bodies [59, 60]. Several of these structures associated with the location of hemozoin formation, the parasite’s digestive vacuole (DV) during the trophozoite stage (Fig. 1e), which is consistent with previous reports [58, 61]. Interestingly, a higher number of lipid bodies were found in gametocytes, where some also were located at the periphery of the cell (Fig. 1f).

The polar lipid probe Nile Red [62] revealed that polar lipids are more enriched at intracellular organelle structures rather than in the parasite plasma membrane. The pattern of membranous structures observed in trophozoites (Fig. 1h), however, was quite different from the more fragmented pattern in gametocytes (Fig. 1I).

Altogether, the microscopic analyses revealed significant differences in morphology, intracellular content and lipid distribution in the RBCs infected with *P. falciparum* gametocytes in comparison to uninfected and trophozoite-infected RBCs (Fig. 1) and prompted us to investigate the detailed lipid composition of RBCs infected with different life-cycle stages of *P. falciparum*.

### The lipid composition changes significantly during sexual development of *P. falciparum*

Malaria parasite infection modifies the lipid composition of the host cells, particularly the plasma membrane [11, 26, 63, 64]. The lipid labelling suggested a marked difference in lipid composition of RBCs infected with the sexual parasites of *P. falciparum* compared with uninfected and asexual trophozoite-infected RBCs. To examine the transformation in lipid composition during development of gametocytes, a detailed lipid profile of RBCs infected with the five sexual stages of *P. falciparum* was generated by mass spectrometry and compared to that of uninfected and trophozoite-infected RBCs. In this study RBCs infected with mid-stage trophozoites (~24–28 h) were chosen as a representative of the asexual stages. A total of 154 lipid molecular species belonging to 10 major lipid classes were identified in whole cell extracts. A detailed summary of quantitative values as well as corresponding proportions compared to the total lipid amounts (in parentheses) of the major lipid classes found in RBCs infected with different parasite stages is shown in Additional file 4.

**Fig. 2:**
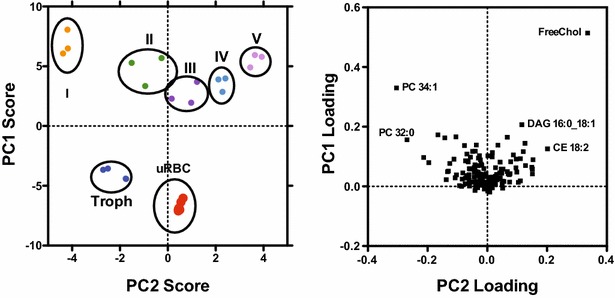
Two-dimensional principal component analysis (PCA) of the uninfected, trophozoite-infected and gametocyte-infected RBCs based on their lipid composition. PCA results were plotted in two-dimensional plots that reflect the behaviour of the RBCs infected with different blood-stage parasites (*score plot*, *left graph*) corresponding to the molecular lipid species (*loadings plot*, *right graph*). *Ovals* in the scores plot group the same samples of three independent experiments. *uRBC* uninfected red blood cells, *Troph* trophozoites, *I–V* gametocytes stage I to V, *DAG* diacylglycerol, *FC* free cholesterol, *PC* phosphatidylcholine, *PE* phosphatidylethanolamine, *PS* phosphatidylserine, *SM* sphingomyelin. *PC1* first principal component, *PC2* second principal component

To depict the global relationship between the cells and the abundance of their lipids, and to elucidate major variation and co-variation patterns between the cells based on their lipid compositions, the obtained lipid data were subject to a principal component analysis (PCA) (Fig. 2). Uninfected RBCs, trophozoite-infected RBCs and gametocyte-infected RBCs could be distinguished based on PCA score plots generated from their lipid profiles (Fig. 2). The loading plot corresponds to the lipids that are a major contributor to the shift in the 2D space [the dimensions are labelled principal component 1 (PC1) and (PC2)]. Here, differences in free cholesterol (FC), PC 34:1 and DAG 16:0_18:1 appear to be related mostly to the PC1 whereas distinctions in FC, PC 32:0, PC 34:1 and CE18:2 were greatly responsible for the PC2. In crude terms PC1 correlates with infection, whereas PC2 correlates with the maturity of the gametocyte infected RBCs. The PCA reveals that the lipid composition of parasite-infected RBCs dramatically alters during sexual development of *P. falciparum*, suggesting alterations in host cell membrane properties and also indicating both significant morphological and metabolic differences in red blood cells infected with different gametocyte stages.

**Fig. 3:**
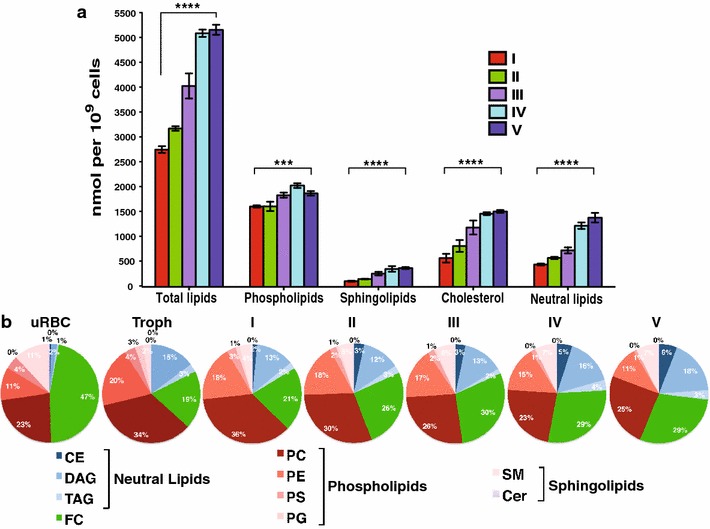
Distinct patterns of lipids in different blood-stage *P. falciparum*. Absolute amounts of major lipid classes in RBCs infected with gametocyte-stage *P. falciparum* (**a**) and proportion of major lipid species (**b**). *CE* cholesteryl ester, *DAG* diacylglycerol, *TAG* triacylglycerol, *FC* free cholesterol, *PC* phosphatidylcholine, *PE* phosphatidylethanolamine, *PS* phosphatidylserine, *PG* phosphatidylglycerol, *SM* sphingomyelin, *Cer* ceramide. *uRBC* uninfected red blood cells, *Troph* trophozoites, *I–V* gametocytes stage I to V. Means and standard deviations of three independent experiments were shown. To determine differences in lipid content of stage I to stage V gametocytes-infected red blood cells, unpaired Student’s t test was performed using GraphPad Prism 6.0. *p < 0.05; **p < 0.01; ***p < 0.001; ****p < 0.0001

RBCs containing mature gametocytes showed more than six times as much total lipid as uninfected cells whereas the total lipid content in trophozoite infected RBCs almost triples compared to uninfected RBCs [see Additional file 4]. Neutral lipids especially contribute to this increase in total lipids during *P. falciparum* gametocyte development (60-fold compared to uninfected RBCs). The proportion of neutral lipids compared to total lipids increases considerably from 3 % in uninfected RBCs to 18 % in trophozoite-infected RBCs consistent with previous reports [58, 61], and amounting to 27 % in mature gametocyte-infected RBCs (Fig. 3b). The change in phospholipids was less dramatic, but still substantial: relative to the uninfected RBCs the increase in phospholipids amounts in trophozoite-infected and gametocyte-infected RBCs were 4.8- and 6.9-fold, respectively, which might reflect the extensive proliferation of parasite membranous system [see Additional file 4]. However, when represented as proportional values, phospholipids decrease continuously from 60 % in trophozoite-infected cells to 58 % in stage I gametocyte and 36 % in stage V gametocytes. In uninfected cells phospholipids contributed 38 % to the total lipid amount (Fig. 3b).

### Cholesterol and sphingolipids decrease upon RBC infection, but increase during gametocyte maturation

Apart from phospholipids and sphingolipids, cholesterol is a major lipid of cellular membranes [65]. The total amount of cholesterol fluctuates notably between RBCs infected with different gametocyte stages: cholesterol accumulated gradually in the infected cells. While its proportion in trophozoite-infected cells was only 19 %, it represented 47 and 29 % in uninfected and late stage gametocyte-infected cells, respectively.

Similar to cholesterol, sphingomyelin was significantly depleted in RBCs infected with trophozoites (p > 0.001) accounting for only 2 % of the total lipids whereas in the uninfected RBCs and mature gametocyte-infected RBCs this lipid represented up to 11 and 7 % of the total lipids, respectively (Fig. 3b). The major SM species in mature gametocyte-infected RBCs contained C16:0, C24:0 or C24:1 acyl chain [see Additional file 5A].

### Phospholipids have the highest relative abundance in infected red blood cells

**Fig. 4:**
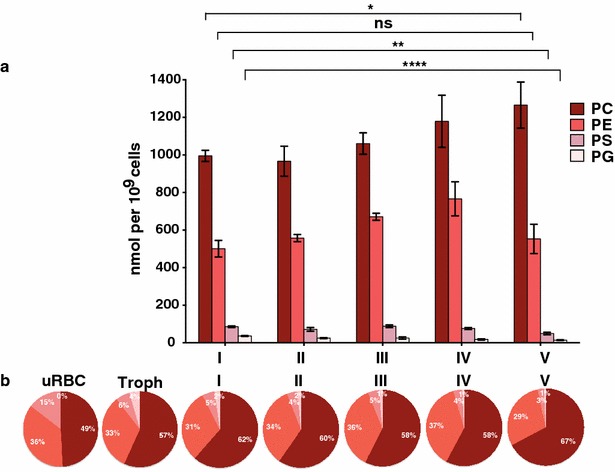
Changes in profile of major phospholipid species during development of *P. falciparum* gametocytes. **a** Phospholipid profiles of red blood cells infected with different *P. falciparum* gametocyte stages. *PC* phosphatidylcholine, *PE* phosphatidylethanolamine, *PS* phosphatidylserine, *PG* phosphatidylglycerol, *uRBC* uninfected red blood cells, *Troph* trophozoites, *I–V* gametocytes stage I to V. Means and standard deviations of three independent experiments were shown. Unpaired Student’s t test was performed using GraphPad Prism 6.0. ns, not significant; *p < 0.05; **p < 0.01; ***p < 0.001; ****p < 0.0001. **b** Proportion of phospholipid groups in uninfected RBCs and RBCs infected with blood-stage *P. falciparum* stages

Unlike uninfected RBCs (where FC is the most abundant lipid species), phospholipids make up the majority of lipids in infected RBCs (Fig. 3b). The amount of total phospholipids increased almost fivefold upon infection of the RBCs with the parasite and increase even more during gametocyte development [see Additional file 4]. This overall increase was mirrored in the total increase of PC and PE, as they were the major components representing more than 90 % of phospholipids in all the examined cell extracts (Fig. 4). Phospholipid species that showed a particular high increase during gametocyte development were PC 34:0, PC 34:2, and PE36:4 [see Additional file 5C, D].

In contrast to PC and PE, PS and PG were less abundant in both uRBC and iRBC. There was a significant drop in relative PS amounts in RBCs infected with trophozoites (reducing from 15 to 6 % of total phospholipids) (Fig. 4); the amount decreased even further upon gametocyte maturation (p > 0.01 comparing absolute amounts in RBCs infected with stage I and stage V gametocytes). On the other hand, PG levels in the RBC increased upon infection, rising from below detection levels to 4 % of the total phospholipids (Fig. 4b). Upon gametocyte maturation the amount of PG decreased again (from 4 to 1 %, p > 0.0001). As in the case of the more abundant phospholipid groups in trophozoite-infected RBCs, where mono-unsaturated species like PC 34:1 and PE 34:1 were one of the most prominent, the mono-unsaturated PG 16:0_18:1 was the most abundant PG species [see Additional file 5F].

Taken together, the substantial difference in phospholipid composition both in absolute and relative numbers between trophozoite-infected and gametocyte-infected cells contribute to the unique lipid profile of the individual stages.

### Neutral lipids are highly enriched in gametocytes

**Fig. 5:**
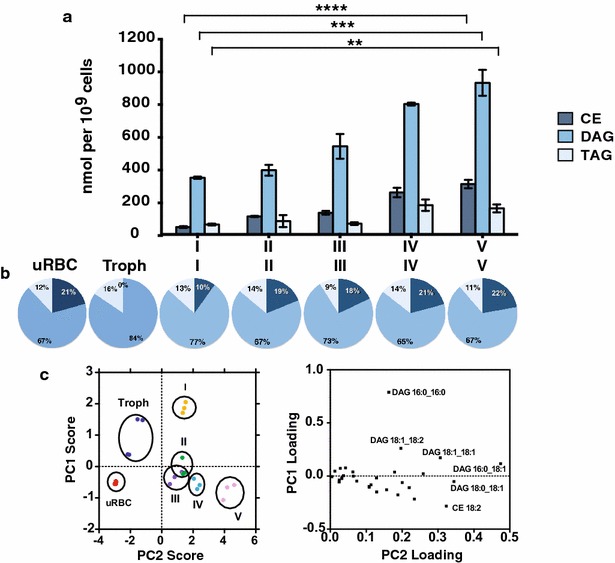
Changes in profile of neutral lipid species during development of *P. falciparum* gametocytes. **a** Neutral lipid profiles of red blood cells infected with different *P. falciparum* gametocyte stages. *CE* cholesteryl ester, *DAG* diacylglycerol, *TAG* triacylglycerol. Means and standard deviations of three independent experiments were shown. Unpaired Student’s t test was performed using GraphPad Prism 6.0. **p < 0.01; ***p < 0.001; ****p < 0.0001. **b** Proportion of neutral lipid groups in RBCs infected with blood-stage *P. falciparum* stages. **c** Two-dimensional principal component analysis (PCA) of uninfected, trophozoite-infected and gametocyte-infected RBCs based on their neutral lipid composition. PCA results were plotted in two-dimensional plots that reflect the behaviour of the RBCs infected with different blood stage parasites (*score plot*, *left graph*) corresponding to the molecular lipid species (*loadings plot*, *right graph*). Ovals in the scores plot group corresponding samples of three independent experiments. *uRBC* uninfected red blood cells, *Troph* trophozoites, *I–V* gametocytes stage I to V

Cholesteryl ester (CE), diacylglycerol (DAG) and triacylglycerol (TAG) are the major neutral lipid classes in eukaryotic cells [66]. Along with the increase in the amount of cholesterol in gametocytes, CE also accumulated substantially in the sexual stage infected RBC starting from 45 × 10^−9^ nM/cell in stage I gametocytes and increased to 300 × 10^−9^ nM/cell in stage V gametocytes (Fig. 5a) (p > 0.0001, unpaired Student’s t-test), representing 2 and 6 % of total lipids, respectively (Fig. 3). These amounts detected in gametocytes were in stark contrast to the CE amounts in uninfected and trophozoite-infected RBCs, which were around the detection limit. The major molecular species of CE contained a di-unsaturated C18:2 acyl chain [see Additional file 5G], indicating a requirement of gametocytes for linoleic acid (C18:2).

Accumulation of DAG has been reported in the mature asexual parasites [55, 58]. Coherent with these findings, a ~sevenfold increase in DAG proportion in the trophozoite-infected RBCs in comparison to uRBCs was detected [from 2 to 15 % (Fig. 3b)]. The DAG amount increased even further in gametocytes: 18 % of total lipids in stage V gametocytes infected RBCs were DAG (Fig. 5a). Trophozoites and stage I gametocyte infected RBCS were especially rich in saturated DAG C16:0_C16:0 species, whereas mature gametocytes contained the highest enrichments for mono-unsaturated C16:0_C18:1 and C18:0_C18:1 acyl chains of all molecular lipid species (Fig. 5c and Additional file 5H).

Overall, DAG showed a similar pattern in abundance as phospholipids. DAG is a central class serving also as precursor for the synthesis of most phospholipids [67] and is converted to TAG through an acyl-CoA:diacylglycerol acyltransferase (DGAT)-mediated pathway (PFC0995c) [55, 60]. The expression of DGAT is highest in late trophozoites and stage V gametocytes. Consistent with this TAG was especially enriched in trophozoites and mature gametocytes, accounting for approximately 3–4 % of total lipids. The most abundant TAG species in trophozoites was mono-unsaturated TAG C50:1 (possibly derived from abundant DAG C16:0_C16:0 and oleic acid [C18:1]), whereas in gametocytes di-unsaturated TAG C52:2 (possibly derived from the most abundant DAG C16:0_C18:1 and oleic acid [C18:1]) was the major species [see Additional file 5I]. Together, the data obtained for neutral lipids indicate that there is a profound accumulation of storage lipids in the iRBCs, resulting in an almost 60-fold increase in mature gametocytes compared to uRBCs.

## Discussion

The presented study shows that lipid composition of parasite-infected RBCs significantly alters during the sexual development of *P. falciparum* and that individual parasite stages can be distinguished by their unique lipid profile. Compared to uninfected RBCs the amount of total lipids increased in trophozoite-infected RBCs 2.5-fold with the majority of lipids accumulating inside the parasite, whereas lipid content more than doubled when the parasite developed to a fully matured gametocyte with significant amounts of lipids additionally distributed across its host cell cytosol and membrane [see Additional file 4]. This overall increase in lipids however is not reflected in all lipid species: the proportion of phospholipids is nearly halved in stage V gametocytes compared to trophozoite-infected RBCs (37 vs 61 %). At the same time neutral lipids, sphingomyelin and cholesterol increase significantly in gametocytes (27 vs 18 %, 7 vs 2 % and 29 vs 19 %, respectively), resulting in a net increase in total lipids. Although below the detection limit in trophozoite stages, CEs rise to considerable levels during gametocyte maturation. Overall, the lipid composition of mature gametocytes is significantly different to the lipid profile of trophozoite-infected RBC.

Previous transcriptome and proteome studies have revealed little in terms of proteins involved in lipid metabolism. However, Silvestrini et al. analysed the proteome of stage I and II gametocytes [68]. These authors identified 25 genes in these stages that are associated with three lipid-specific GO terms (cellular lipid metabolism, lipid metabolic process, lipid biosynthetic process). The overall expression profiles of these genes [69] are consistent with the findings of our studies: in comparison to trophozoite stages the transcripts of biosynthetic enzymes that are involved in the production of phospholipids (like phosphoethanolamine N-methyltransferase or phosphatidylglycerophosphate synthase) are decreased in gametocytes whereas transcripts for enzymes involved in cholesterol synthesis are increased in gametocytes (e.g. steryl ester hydrolase or geranyl geranyl pyrophosphate synthase).

### Lipids involved in iRBC morphology

Morphological changes during gametocyte development include a conversion of a fairly spherical trophozoite infected RBC to an increase in length resulting in the characteristic crescent shape in later stage gametocytes. At the same time the surface area and volume of infected RBCs do not change significantly [20, 70]. Although this transformation is partly driven by structures underneath the host cell membrane (i.e. the inner membrane complex [19] and microtubules [71]), the present analysis suggests a potential involvement of lipids.

The larger proportion of phospholipids (in particular PC) present in trophozoite compared to gametocytes infected RBCs might reflect the need for these lipids in preparation for merozoite membrane production. Female gametocytes do not have this requirement and any additional lipid membranes that might be required in the male gametocyte will be less obvious due to the sex bias towards females.

### Lipids important for the survival in the host

The major mechanisms that prevent immature gametocytes from entering the peripheral circulation seem to be mechanical retention in the bone marrow and the spleen [18]. Maturation of gametocytes is characterized by the transformation of a relatively stiff infected RBC to a deformable late stage gametocyte (similar to uRBCs), which is released into the peripheral circulation of the host in order to facilitate transmission to mosquitoes. The shift in the relative content of cholesterol and SM during the development of *P. falciparum* gametocytes observed in our study is consistent with the change in the deformability of malaria sexual parasites and splenic pass rates [18–20] published recently. The molecular mechanism responsible for the shift from stiff, adherent to deformable, circulating gametocytes is still poorly understood but possibly involves both the synthesis and partial destruction of the inner membrane complex of gametocytes [19] and the membrane lipid composition as described in this study. The changes seen in stage IV can be interpreted as the preparation of the gametocyte for a ride in the circulation, once the stage V gametocytes stop sequestering. Hence, the changes in lipid composition might facilitate the availability of gametocytes for transmission in the peripheral blood.

Cholesterol is a key molecule for the stability of biological membranes, however, excess free cholesterol is toxic for cells [72]. Given the importance of cholesterol homeostasis the fluctuation of cholesterol during the parasite development is remarkable. Membranes of uninfected RBCs are particularly rich in cholesterol (e.g. Fig. 1). There are three possible mechanisms for the observed drop in cholesterol content upon infection: (1) increased metabolism and conversion into other molecules, (2) reduced uptake from the serum and/or (3) increased excretion. The removal of cholesterol by converting it into CE is unlikely, since CE is almost absent in the trophozoite stages.

The predominance of PC, PE and PG molecular species containing mono-unsaturated C34:1 acyl composition in the trophozoite-infected RBCs is consistent with the data shown in a recent report [73] and the finding that the asexual parasites require exogenous supply of oleic acid (C18:1) and palmitic acid (C16:0) for normal growth [74]. The abundance of PC 34:1 and PE 34:1 in gametocytes suggests a similar role for oleic acid and palmitic acid in the development of gametocytes.

Most recently, Gulati et al. [75] provided compelling evidence that lipid classes, which are enriched in certain life-cycle stages (like TAGs and SM), represent excellent drug targets. This is of particular importance given the relative abundance of pharmacological compounds targeting the lipid metabolism already approved for use in humans.

The unique lipid composition, the dependence on key lipid species and the absence of obvious enzyme homologues for lipid conversion might provide new avenues to stop *Plasmodium* infections.

### Lipids involved in storage

Mature gametocytes face profound changes in their environment after they are taken up by the mosquito. In order to avoid being digested with the blood meal and to be able to undergo sexual recombination through the fusion of male and female gametes the parasite has to undergo rapid and far-reaching changes.

Neutral lipids are the main contributor to the substantial increase in overall lipids upon parasite infection and gametocyte maturation. uRBCs contain hardly any storage lipids. They are believed to be mainly present in lipid bodies [57–59]. The accumulation and carry over of essential lipids in the female parasite ensures their availability after transition to and fertilization in the mosquito.

Despite being virtually absent in uninfected and trophozoite-infected RBCs [55], CEs are highly enriched upon gametocyte maturation. The increases in CE levels are likely to contribute to the enhanced formation of neutral lipid bodies in gametocytes. In mammalian cells, CE functions primarily as caloric reserves and as caches of fatty acid and sterol components that are needed for membrane biogenesis [65]. Of note is also that in stage IV gametocyte infected RBCs the ratio of unsaturated to saturated neutral lipids is almost four times higher than in uRBCs [see Additional file 6D]. Mosquitos lack the biochemical pathways to add a second or third double bond into fatty acids nor can they synthesize sterols de novo [76]. Hence access to poly-unsaturated fatty acids and cholesterol might be restricted for the parasite once inside the mosquito.

Along with cholesterol and CEs, other neutral lipids including DAGs and TAGs also accumulate in gametocytes. Apart from CE, TAG is also a major storage lipid present in lipid bodies [59, 66]. The remarkable enrichment of neutral lipids in mature gametocytes may serve as a major energy storage to fuel the sudden increase in protein and phospholipid biosynthesis required during gametogenesis and early zygote development in the insect host when there is limited access to certain lipid species. Competition for lipids is a major factor by which mosquitoes carrying the bacterial endosymbiont *Wolbachia* are better protected against infections with pathogens including *Plasmodium* [77]. Hence understanding lipid acquisition, storage and usage of lipids in *P. falciparum* might lead to novel intervention strategies.

### Lipids as signalling molecules

Signalling events are especially important in the late stages of gametocytogenesis. An increase in lipids potentially acting as signalling molecules in the mature gametocyte is therefore not surprising and might reflect the preparation of this gametocyte stage for activation.

The enrichment of DAG in mature gametocytes may mirror the sensitive state of the cells: profound changes are imminent for the transition to the mosquito such as rounding-up, egress from host cell and differentiation into micro- and macrogametes.

It has been shown previously that ceramides are able to replace cholesterol in microdomains and that this change might facilitate the action of membrane-associated proteins and, therefore, allow cross-talk between different signalling pathways [78, 79]. The significant increase in ceramides in the mature gametocyte stages might reflect the activation of these signalling pathways.

However, secondary messengers are likely to function locally and in a short time window, the resolution of the global lipidomic analysis approach on hand is not sensitive enough to draw definitive conclusions for the role of these lipids in signalling events.

## Conclusions

For the first time a comprehensive analysis of the lipid composition of red blood cells infected with the five different gametocyte stages of *P. falciparum* is presented in this study. The lipid constituents of each individual stage are specific and this information will provide the basis for a better understanding of the parasite’s metabolism. It will also help in the identification and rationalization of drugs that target lipids. Ideally, the analysis would have distinguished between the parasite itself and the surrounding host cell. However, due to the inability to completely separate host from parasite material (since saponin interferes dramatically with the lipids in the membrane) this was not done. In order to investigate the particular low-level lipids, a targeted scanning mass-spectrometry approach with corresponding lipid standards is required.

Since gametocytes take up significant amounts of cholesterol and other lipids from the host cell and/or serum to support their growth, the identification of enzymes involved in lipid metabolic pathways by functional genomics will be significant. Further studies on lipidomics of isolated organelles/subfractions in relation to that of whole parasites as well as using metabolic radioactive labelling may also provide new insights into lipid metabolic pathways of transmissible stages of the malaria parasite. Furthermore, determining the different lipid composition in male and female gametocytes might provide clues about the mechanism of fertilization. The parasite lipidome is a promising area both in terms of uncovering novel biological mechanisms and for the discovery of new drugs to prevent malaria transmission.

## Additional files

10.1186/s12936-016-1130-zQuality control of parasite samples used for lipidomic analysis. A. Giemsa stain of cells before magnet purification. Scale bar, 5 µm. B. Western blot analysis of magnet purified parasites. An antiserum against Pfs16 was used as a marker for gametocytes and an antiserum against PfHSP70 was used as a control for the amount of protein loaded. Troph, trophozoite; I-V, Gametocyte stages I-V.
10.1186/s12936-016-1130-zTarget lipid class, ion detected, internal standard and amount per sample. PC, phosphatidylcholine; PG, phosphatidylglycerol; SM, sphingomyelin; Cer, ceramide; PS, phosphatidylserine; CE, cholesteryl ester; Free Chol, free cholesterol; DAG, diacylglycerol; TAG, triacylglycerol.
10.1186/s12936-016-1130-zTarget lipid class, ion mode, MS/MS experiment (precursor ion (PI) or neutral loss (NL)), and collision-induced dissociation (CID) energy. PC, phosphatidylcholine; PG, phosphatidylglycerol; SM, sphingomyelin; Cer, ceramide; PS, phosphatidylserine; CE, cholesteryl ester; Free Chol, free cholesterol; DAG, diacylglycerol; TAG, triacylglycerol.
10.1186/s12936-016-1130-zContent (nmol / 10^9^ cells, Mean ± SD) and percentage (in parentheses) of the lipid classes and major lipid species of the blood-stage *P. falciparum* infected red blood cells. Parasites were enriched with higher than 95% parasitemia by magnet purification and global lipidomics of whole-cell extracts were analysed by mass spectrometry. uRBC, uninfected red blood cell; I-V, gametocytes stage I to V; n.d., not detected.
10.1186/s12936-016-1130-zContent of molecular lipid species of the blood-stage *P. falciparum* infected red blood cells. A. Sphingomyelin (SM). B. Ceramide (Cer). C. Phosphatidylcholine (PC). D. Phosphatidylethanolamine (PE). E. Phosphatidylserine (PS). F. Phosphatidylglycerol (PG). G. Cholesteryl ester (CE). H. Diacylglycerol (DAG). I. Triacylglycerol (TAG). I-V, gametocytes stage I to V; O, ether-linked lipids. Means and standard deviations of at least three independent experiments are shown.
10.1186/s12936-016-1130-zRatio of unsaturated to saturated lipids in uninfected red blood cells (uRBC), erythrocytes infected with trophozoite-stage parasites (Troph) and erythrocytes infected with stage IV gametocytes (IV). A. Total lipids. B. Phospholipids. C. Sphingolipids. D. Neutral lipids. Means (±S.D.) are shown of three independent samples and compared using unpaired *t*-test. ns, not significant; *, p<0.05; **, p<0.01; ***, p<0.001.
