# Supplementary figures and images for: Changes in lipid composition during sexual development of the malaria parasite Plasmodium falciparum
Source: Malar J. 2016 Feb 6;15:73. doi: 10.1186/s12936-016-1130-z (PMC4744411; doi:10.1186/s12936-016-1130-z)

## Additional file 1

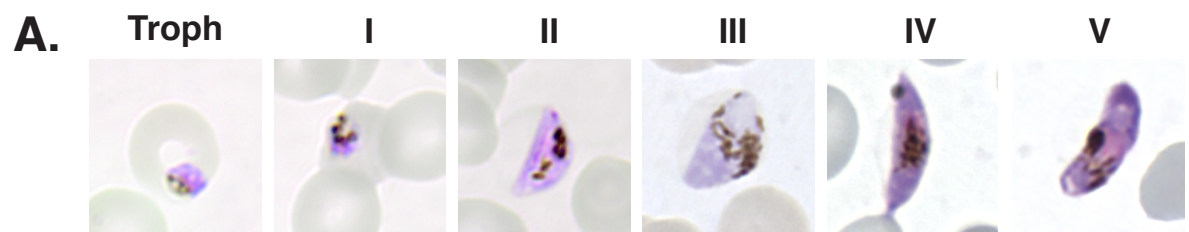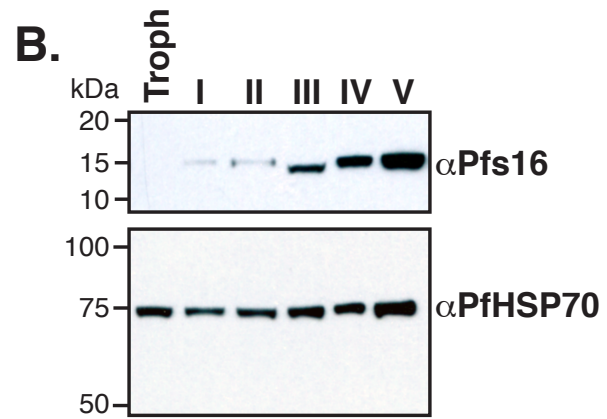

Supplement: Supplementary file 1 — 10.1186/s12936-016-1130-zQuality control of parasite samples used for lipidomic analysis. A. Giemsa stain of cells before magnet purification. Scale bar, 5 µm. B. Western blot analysis of magnet purified parasites. An antiserum against Pfs16 was used as a marker for gametocytes and an antiserum against PfHSP70 was used as a control for the amount of protein loaded. Troph, trophozoite; I-V, Gametocyte stages I-V. [file 12936_2016_1130_MOESM1_ESM.pdf]

Additional file 6

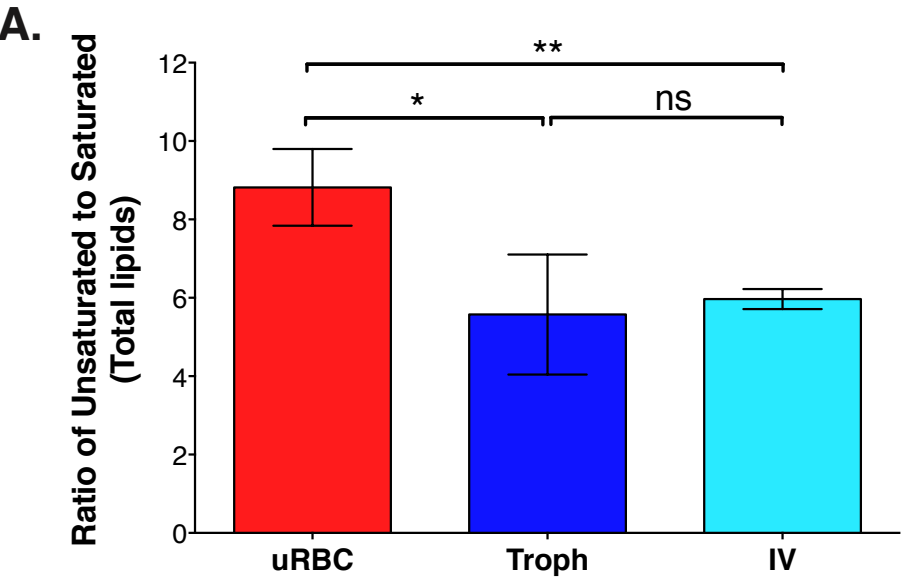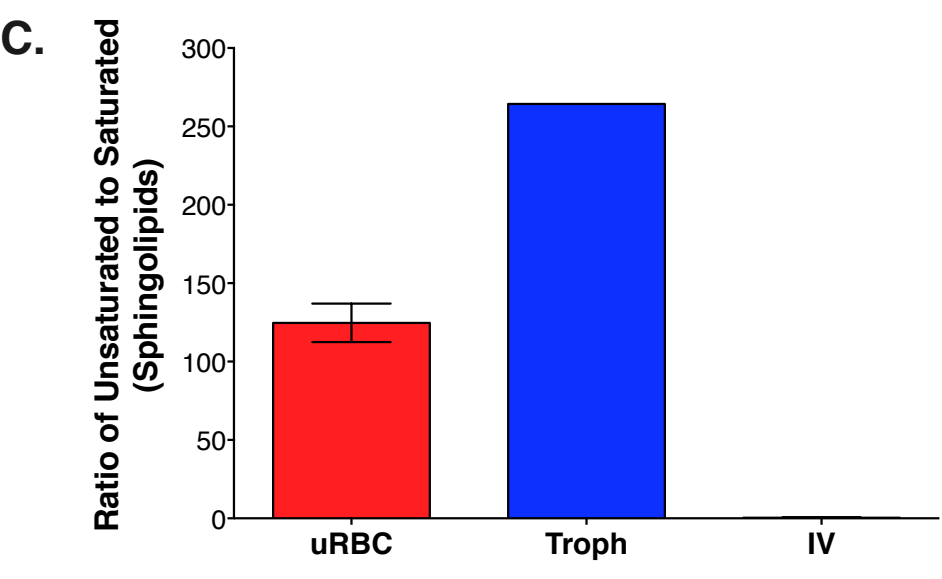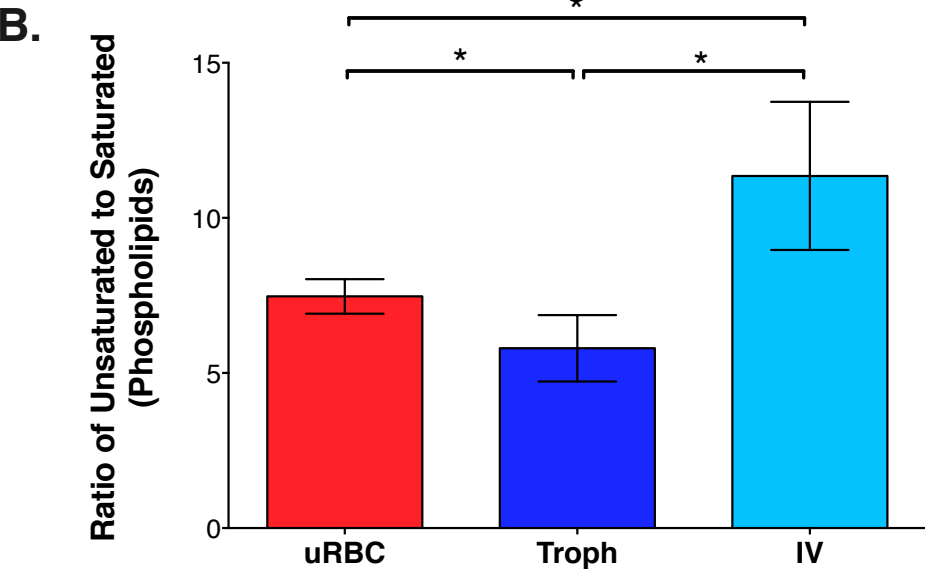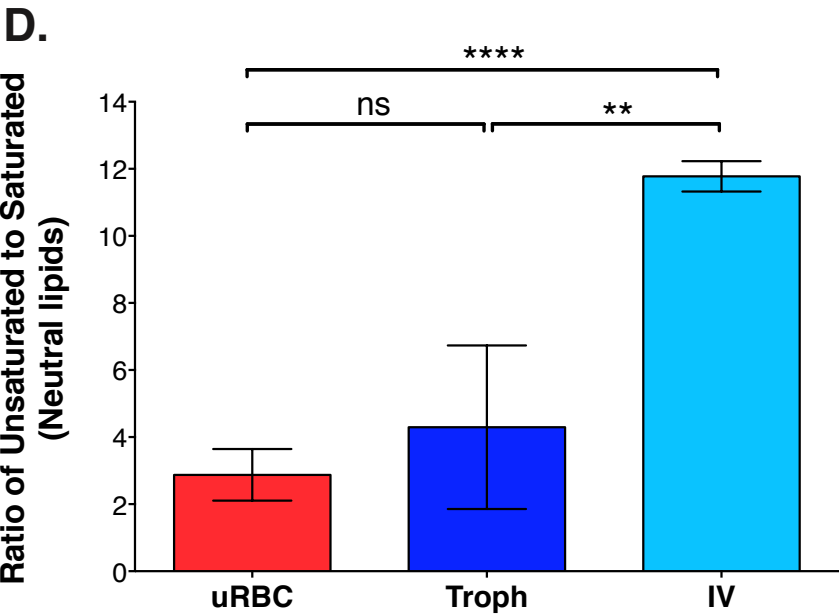

Supplement: Supplementary file 6 — 10.1186/s12936-016-1130-zRatio of unsaturated to saturated lipids in uninfected red blood cells (uRBC), erythrocytes infected with trophozoite-stage parasites (Troph) and erythrocytes infected with stage IV gametocytes (IV). A. Total lipids. B. Phospholipids. C. Sphingolipids. D. Neutral lipids. Means (±S.D.) are shown of three independent samples and compared using unpaired t-test. ns, not significant; *, p<0.05; **, p<0.01; ***, p<0.001. [file 12936_2016_1130_MOESM6_ESM.pdf]
