# Supplementary material for: Changes in lipid composition during sexual development of the malaria parasite Plasmodium falciparum
Source: Malar J. 2016 Feb 6;15:73. doi: 10.1186/s12936-016-1130-z (PMC4744411; doi:10.1186/s12936-016-1130-z)
Supplement: Supplementary file 2 — 10.1186/s12936-016-1130-zTarget lipid class, ion detected, internal standard and amount per sample. PC, phosphatidylcholine; PG, phosphatidylglycerol; SM, sphingomyelin; Cer, ceramide; PS, phosphatidylserine; CE, cholesteryl ester; Free Chol, free cholesterol; DAG, diacylglycerol; TAG, triacylglycerol. [file 12936_2016_1130_MOESM2_ESM.pdf]

Additional file 2.

Lipid Class and Ion	Internal Standard	nmol/sample
PC $[M+H]^+$	PC 19:0_19:0	8
PG $[M-H]^-$	PG 17:0_17_0	1
PE $[M+H]^+$	PE 17:0_17:0	5
SM $[M+H]^+$	DHSM 12:0	2
Cer $[M+H]^+$	Cer 17:0	1
PS $[M+H]^+$	PS 17:0_17:0	1
CE $[M+NH_4]^+$	CE 22:1	1
Free Chol $[M+NH_4]^+$	D_7 Free Chol	10
DAG $[M+NH_4]^+$	DAG 17:0_17:0	1
TAG $[M+NH_4]^+$	D_5 TAG 16:0_16:0_16:0	1
