# Supplementary material for: Changes in lipid composition during sexual development of the malaria parasite Plasmodium falciparum
Source: Malar J. 2016 Feb 6;15:73. doi: 10.1186/s12936-016-1130-z (PMC4744411; doi:10.1186/s12936-016-1130-z)
Supplement: Supplementary file 3 — 10.1186/s12936-016-1130-zTarget lipid class, ion mode, MS/MS experiment (precursor ion (PI) or neutral loss (NL)), and collision-induced dissociation (CID) energy. PC, phosphatidylcholine; PG, phosphatidylglycerol; SM, sphingomyelin; Cer, ceramide; PS, phosphatidylserine; CE, cholesteryl ester; Free Chol, free cholesterol; DAG, diacylglycerol; TAG, triacylglycerol. [file 12936_2016_1130_MOESM3_ESM.pdf]

Additional file 3.

Target Lipid	Ion mode	MS/MS	CID energy
**PC**	+ ve	PI 184.1 *m/z*	40
**PG**	- ve	PI fatty acids	40-55
**SM**	+ ve	PI 184.1 *m/z*	40
**Cer**	+ ve	PI 264.4 *m/z*	35
**PE**	+ ve	NL 141	30
**PS**	+ ve	NL 185	25
**CE**	+ ve	PI 369.4 *m/z*	25
**Free Chol**	+ ve	PI 369.4 *m/z*	15
**TAG**			
**14:0**	+ ve	NL 245.2	35
**16:1**	+ ve	NL 271.3	35
**16:0**	+ ve	NL 273.3	35
**18:2**	+ ve	NL 297.3	35
**18:1**	+ ve	NL 299.3	35
**18:0**	+ ve	NL 301.3	35
**20:4**	+ ve	NL 321.3	35
**22:6**	+ ve	NL 345.3	35
**DAG**			
**16:1**	+ ve	PI 311.3 *m/z*	32
**16:0**	+ ve	PI 313.3 *m/z*	32
**17:0**	+ ve	PI 327.3 *m/z*	32
**18:2**	+ ve	PI 337.3 *m/z*	32
**18:1**	+ ve	PI 339.3 *m/z*	32
**18:0**	+ ve	PI 341.3 *m/z*	32
