# Supplementary material for: Changes in lipid composition during sexual development of the malaria parasite Plasmodium falciparum
Source: Malar J. 2016 Feb 6;15:73. doi: 10.1186/s12936-016-1130-z (PMC4744411; doi:10.1186/s12936-016-1130-z)
Supplement: Supplementary file 4 — 10.1186/s12936-016-1130-zContent (nmol / 109 cells, Mean ± SD) and percentage (in parentheses) of the lipid classes and major lipid species of the blood-stage P. falciparum infected red blood cells. Parasites were enriched with higher than 95% parasitemia by magnet purification and global lipidomics of whole-cell extracts were analysed by mass spectrometry. uRBC, uninfected red blood cell; I-V, gametocytes stage I to V; n.d., not detected. [file 12936_2016_1130_MOESM4_ESM.pdf]

Additional file 4.

Lipid	uRBC		Trophozoite		I		II		III		IV		V	
**Phospholipids**														
phosphatidylcholine	181 ± 27	(23)	741 ± 240	(34)	995 ± 17	(36)	967 ± 46	(30)	1060 ± 33	(26)	1179 ± 80	(23)	1265 ± 71	(24)
phosphatidylethanolamine	86 ± 14	(11)	425 ± 150	(20)	501 ± 25	(18)	557 ± 11	(17)	671 ± 11	(17)	766 ± 52	(15)	553 ± 45	(11)
phosphatidylserine	4.3 ± 6.7	(4.3)	79.7 ± 32.6	(3.7)	85.7 ± 2.6	(3.1)	71.3 ± 5.9	(2.2)	88.2 ± 3.9	(2.2)	76.0 ± 2.8	(1.5)	49.4 ± 3.7	(1.0)
phosphatidylglycerol	0.2 ± 0.2	(0.0)	58.5 ± 25.0	(2.7)	36.2 ± 1.0	(1.3)	24.9 ± 1.6	(0.8)	25.5 ± 3.4	(0.6)	18.0 ± 2.0	(0.4)	14.3 ± 0.8	(0.3)
**Sphingolipids**														
sphingomyelin	89 ± 15	(11)	52.2 ± 18.1	(2.4)	108.4 ± 5.4	(3.9)	151.7 ± 3.1	(4.8)	255 ± 20	(6.3)	346 ± 32	(6.8)	362 ± 11	(7.0)
ceramide	4.9 ± 0.6	(0.6)	5.5 ± 3.3	(0.3)	6.8 ± 0.1	(0.2)	7.8 ± 0.2	(0.2)	12.2 ± 1.2	(0.3)	15.1 ± 1.5	(0.3)	18.1 ± 1.1	(0.3)
**Cholesterol**	366 ± 62	(47)	410 ± 9	(19)	579 ± 51	(21)	823 ± 68	(26)	1195 ± 81	(30)	1473 ± 16	(29)	1518 ± 16	(29)
**Neutral lipids**														
cholesteryl esters	2.9 ± 1.1	(0.3)	n.d.		44.8 ± 3.3	(1.6)	109.6 ± 2.1	(3.4)	132.1 ± 6.8	(3.3)	256 ± 17	(5.0)	308 ± 15	(5.9)
diacylglycerols	16 ± 2	(2.0)	319.4 ± 100	(15)	346.3 ± 3.8	(13)	392 ± 19	(12)	538 ± 44	(13)	796.2 ± 5.2	(16)	926 ± 46	(18)
triacylglycerols	4.7 ± 0.4	(0.6)	59 ± 22	(2.7)	60.3 ± 3.2	(2.2)	81 ± 21	(2.5)	66.0 ± 4.8	(1.6)	178 ± 20	(3.5)	159 ± 14	(3.1)
**Total lipids**	784 ± 125		2151 ± 576		2763 ± 38		3186 ± 26		4043 ± 145		5103 ± 43		5172 ± 59	
**Cholesterol / Phospholipids**	1.22		0.31		0.34		0.47		0.57		0.62		0.68	
