# Supplementary material for: Changes in lipid composition during sexual development of the malaria parasite Plasmodium falciparum
Source: Malar J. 2016 Feb 6;15:73. doi: 10.1186/s12936-016-1130-z (PMC4744411; doi:10.1186/s12936-016-1130-z)

Additional file 5A.

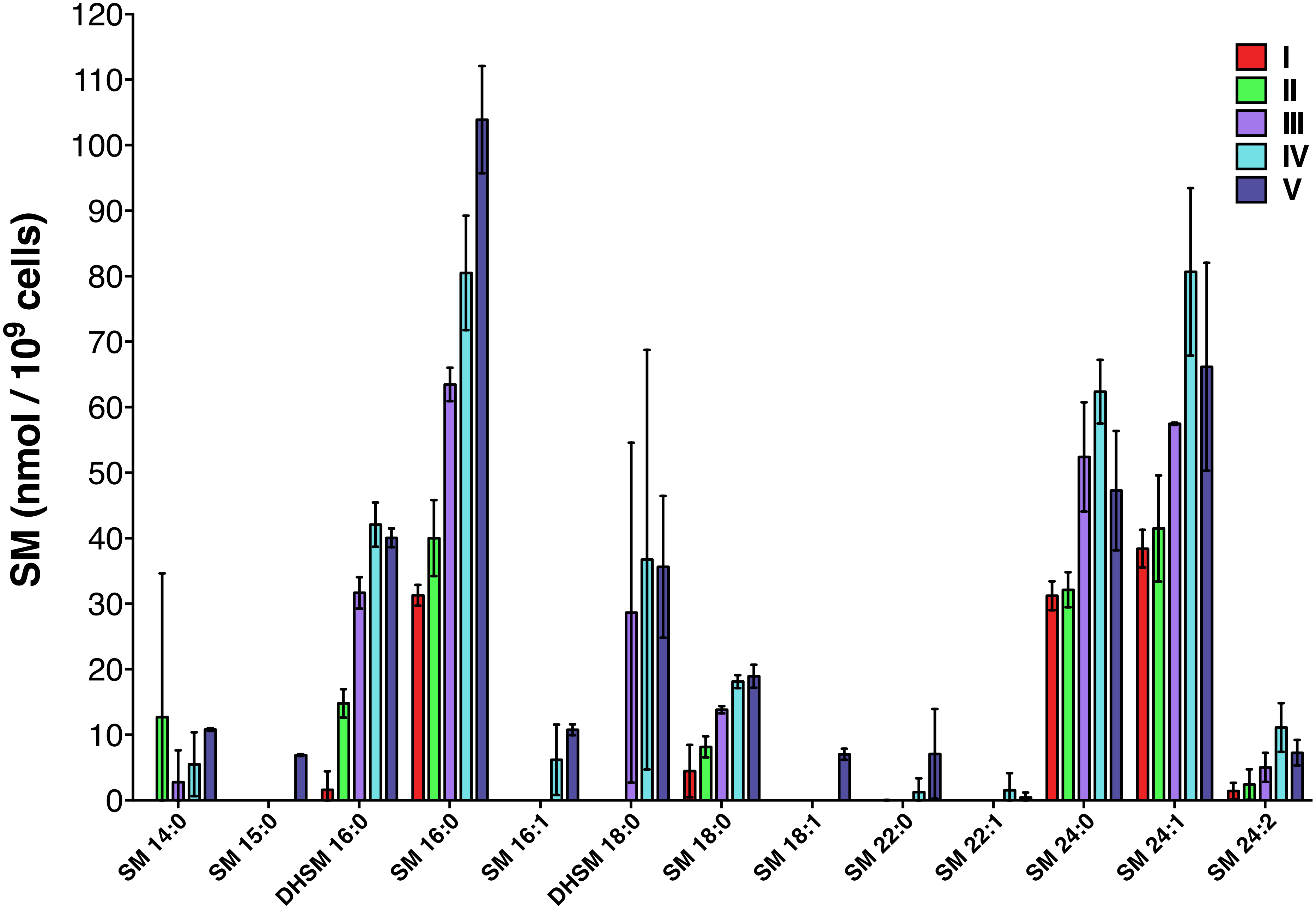

Additional file 5B.

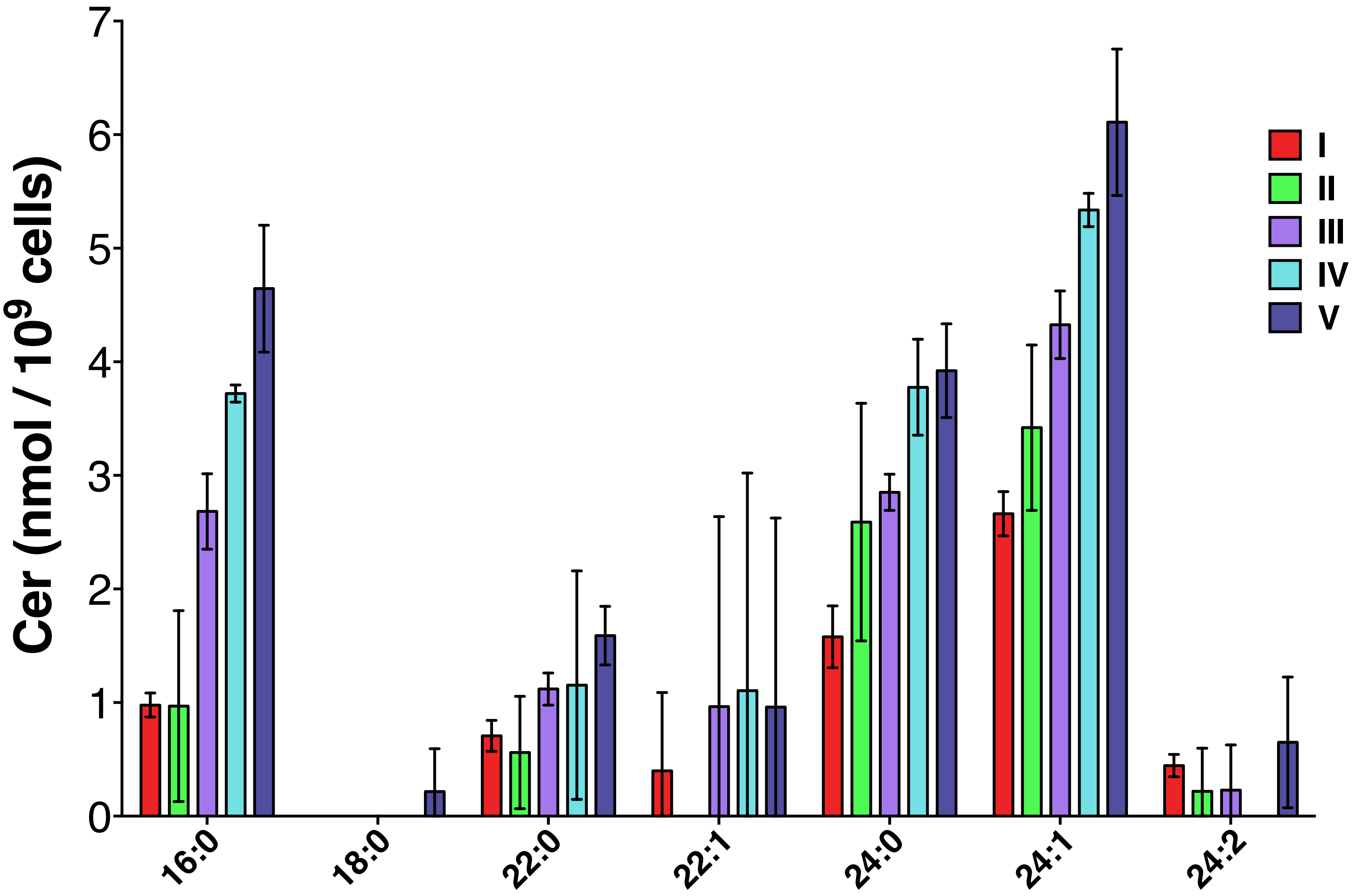

Additional file 5C.

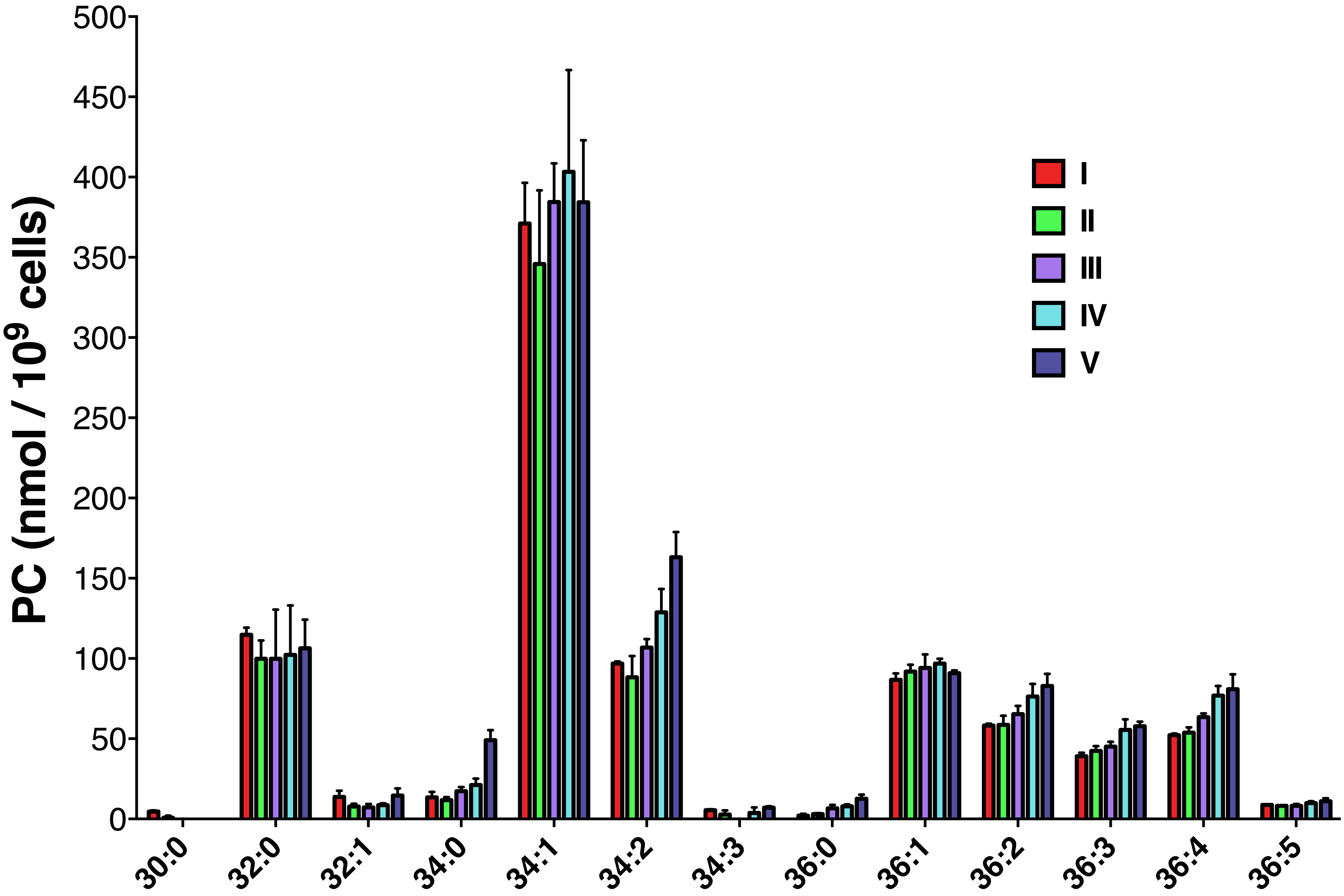

Additional file 5C. (cont)

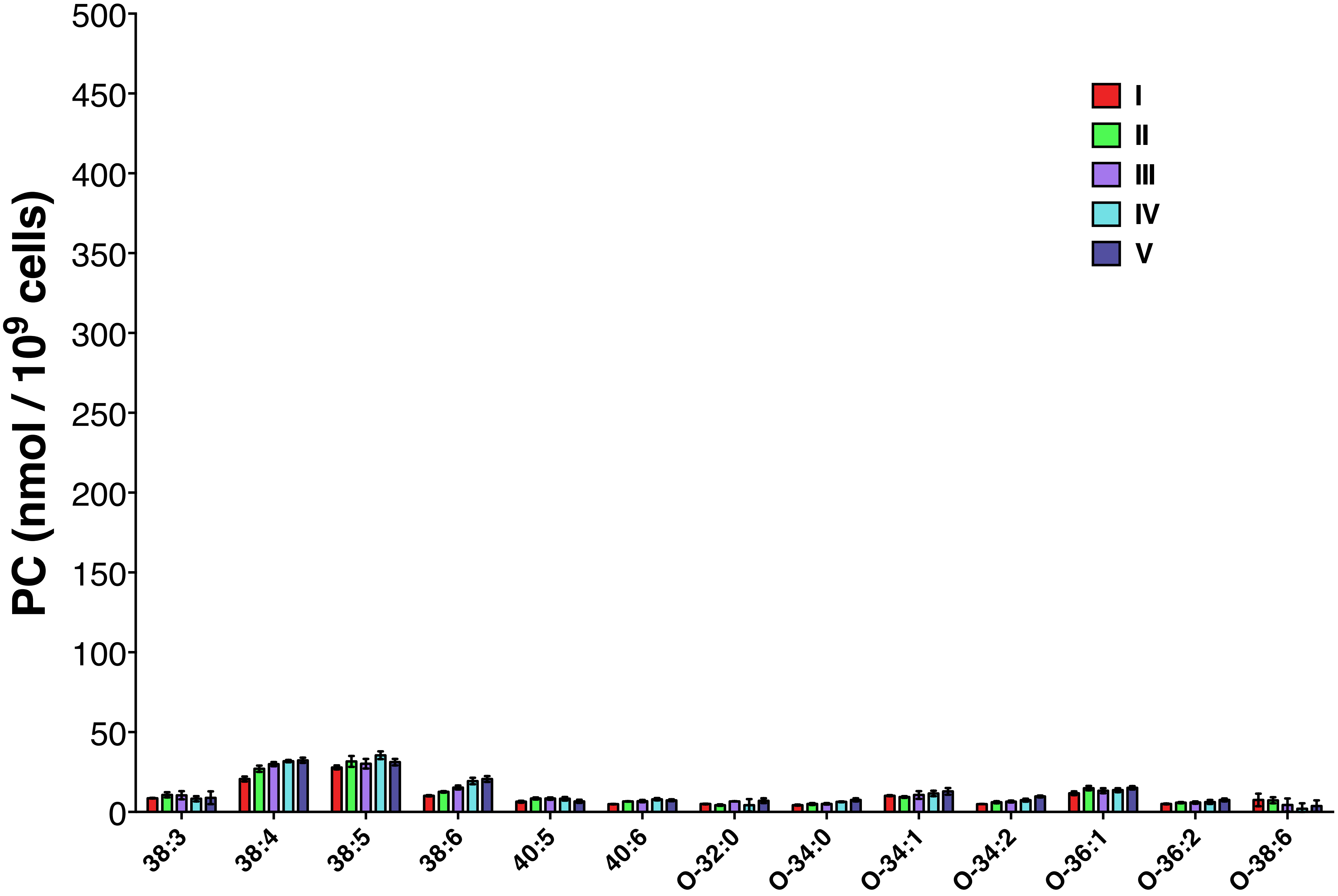

Additional file 5D.

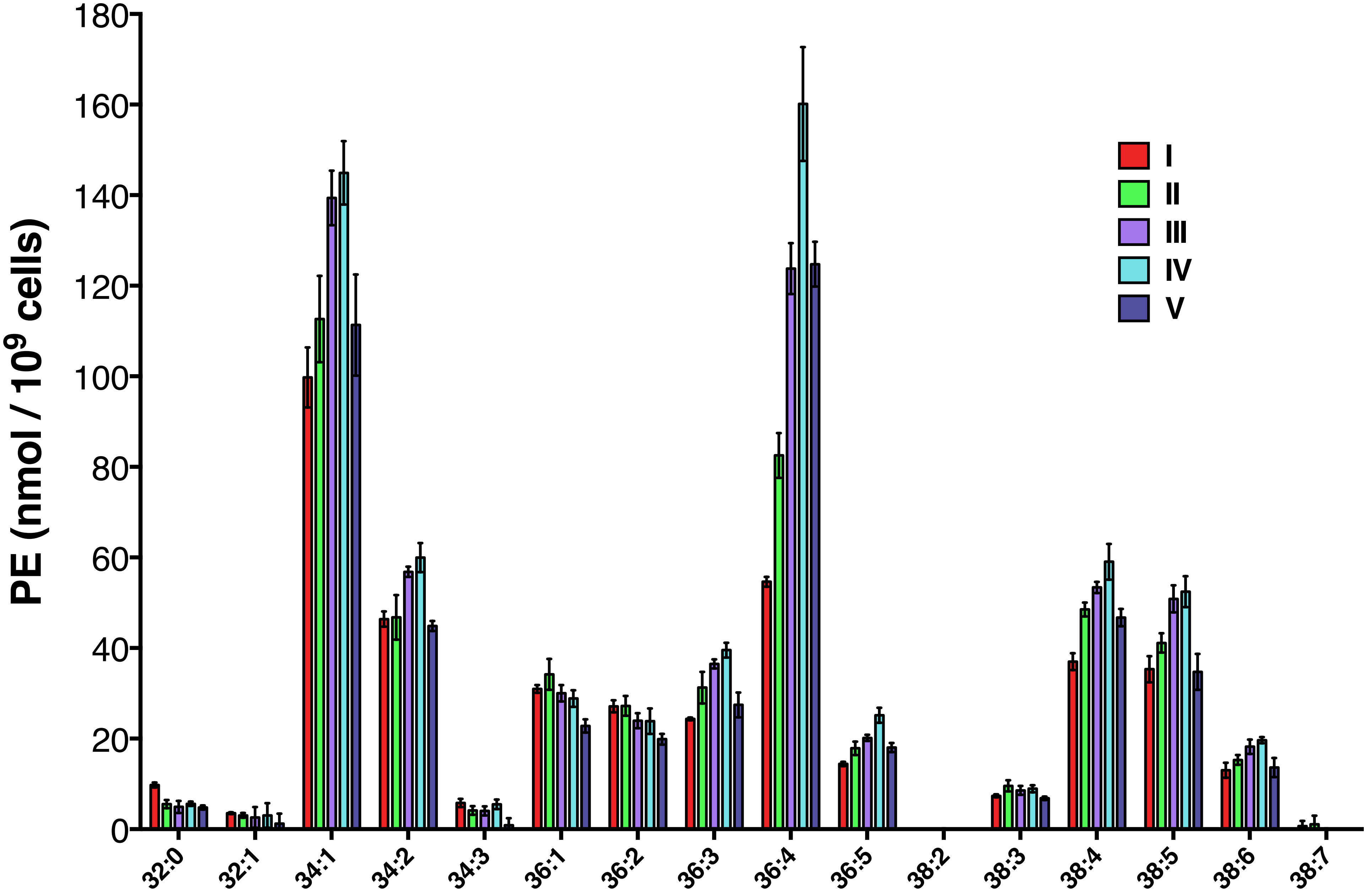

Additional file 5D. (cont)

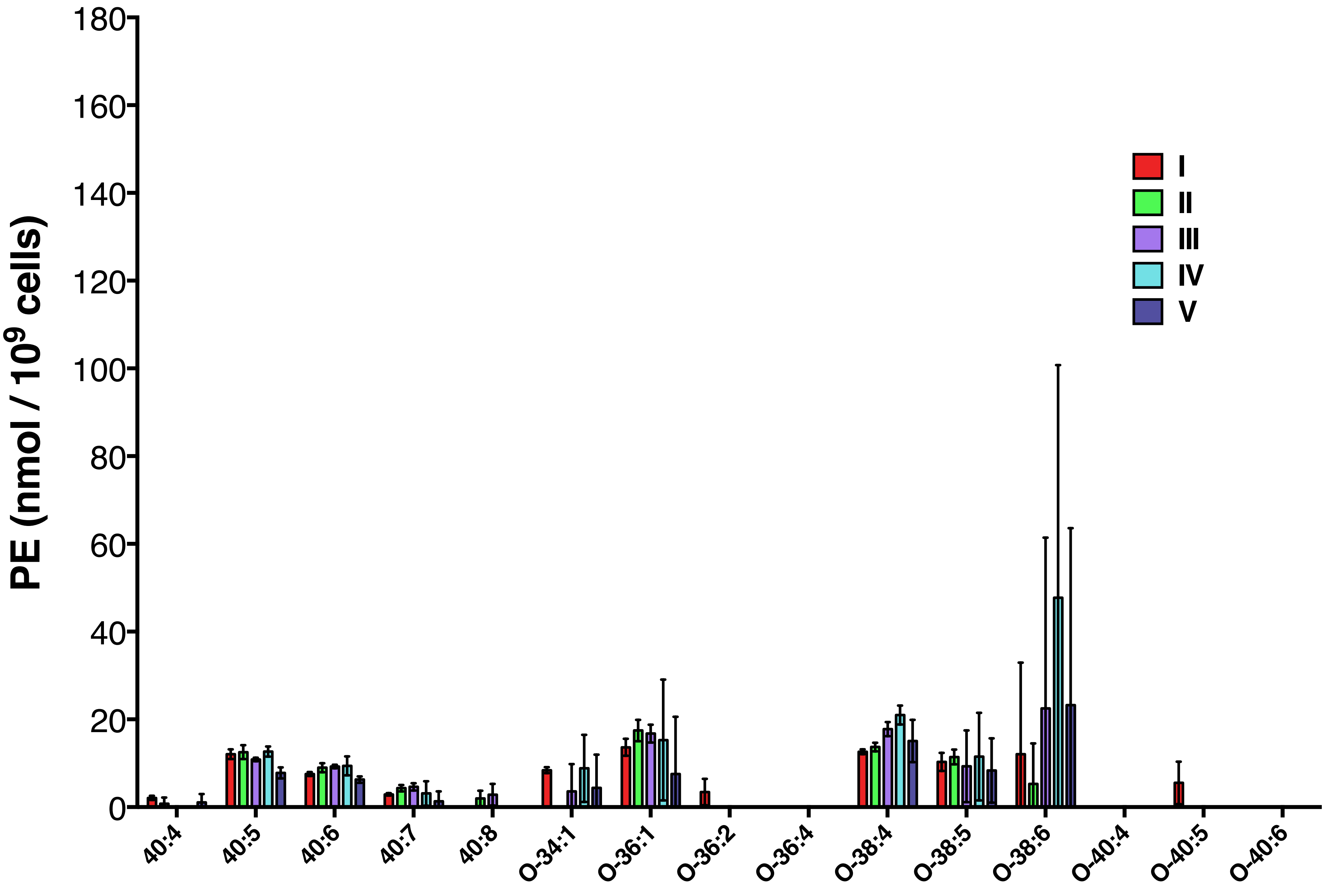

Additional file 5E.

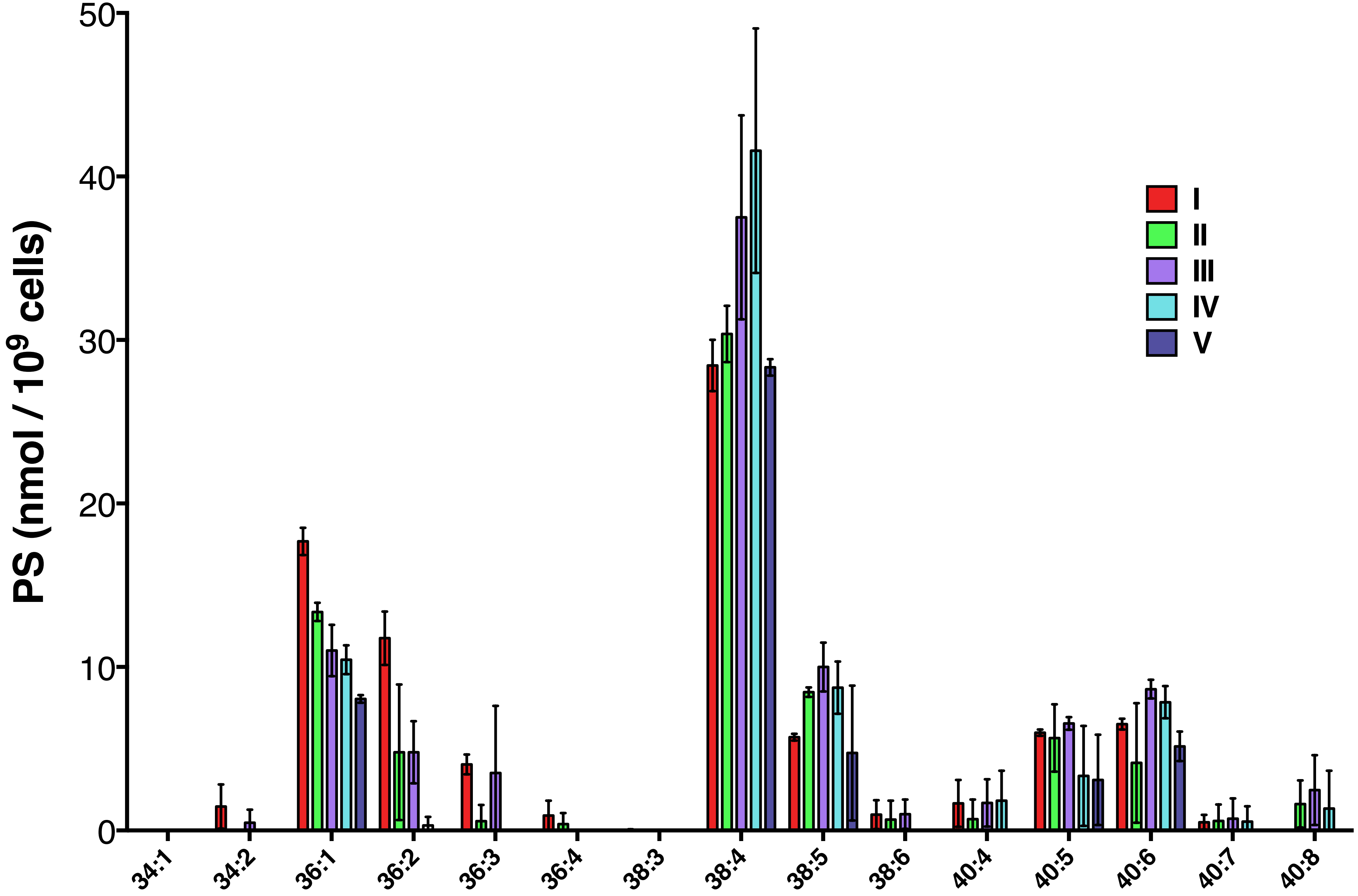

Additional file 5F.

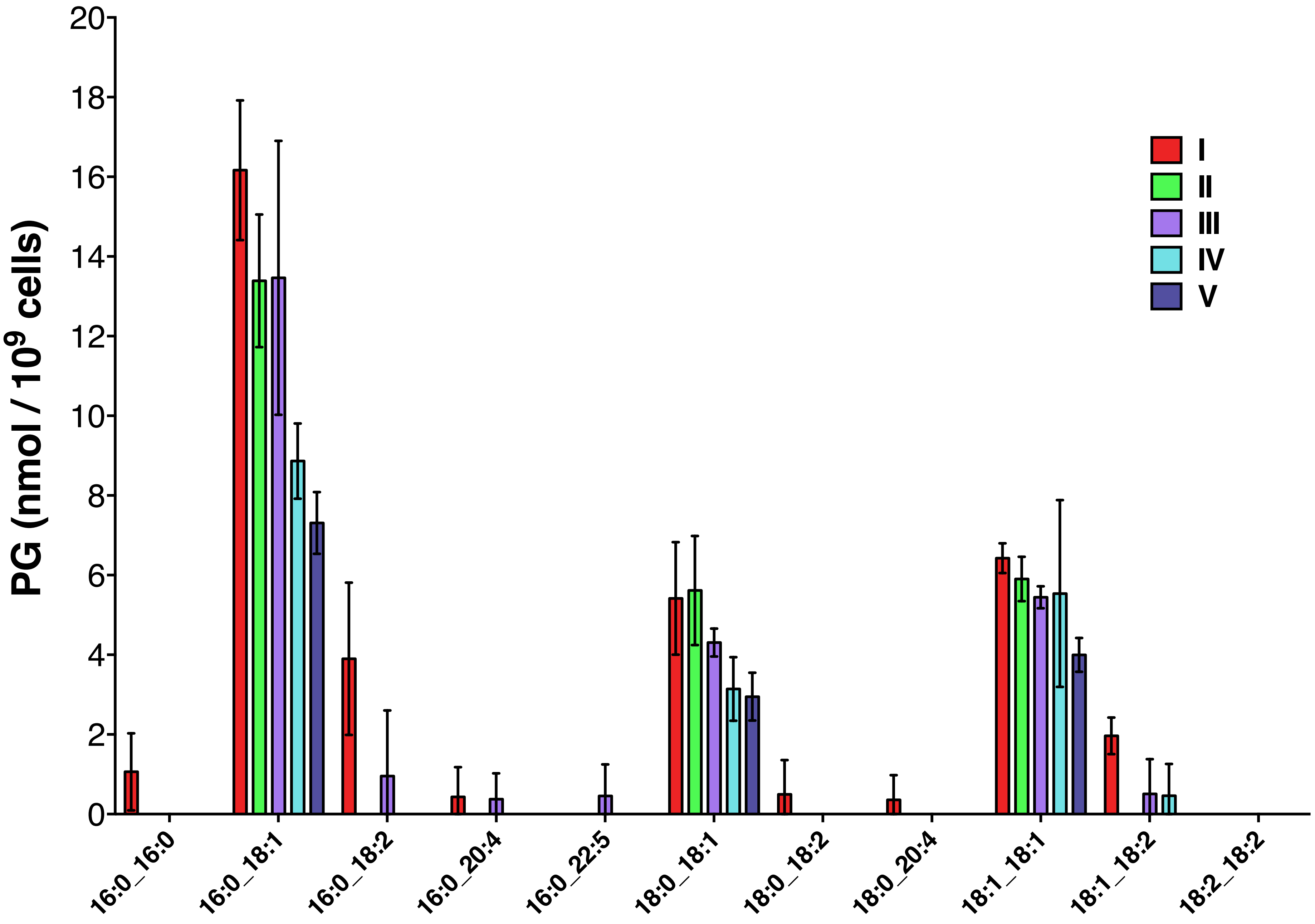

Additional file 5G.

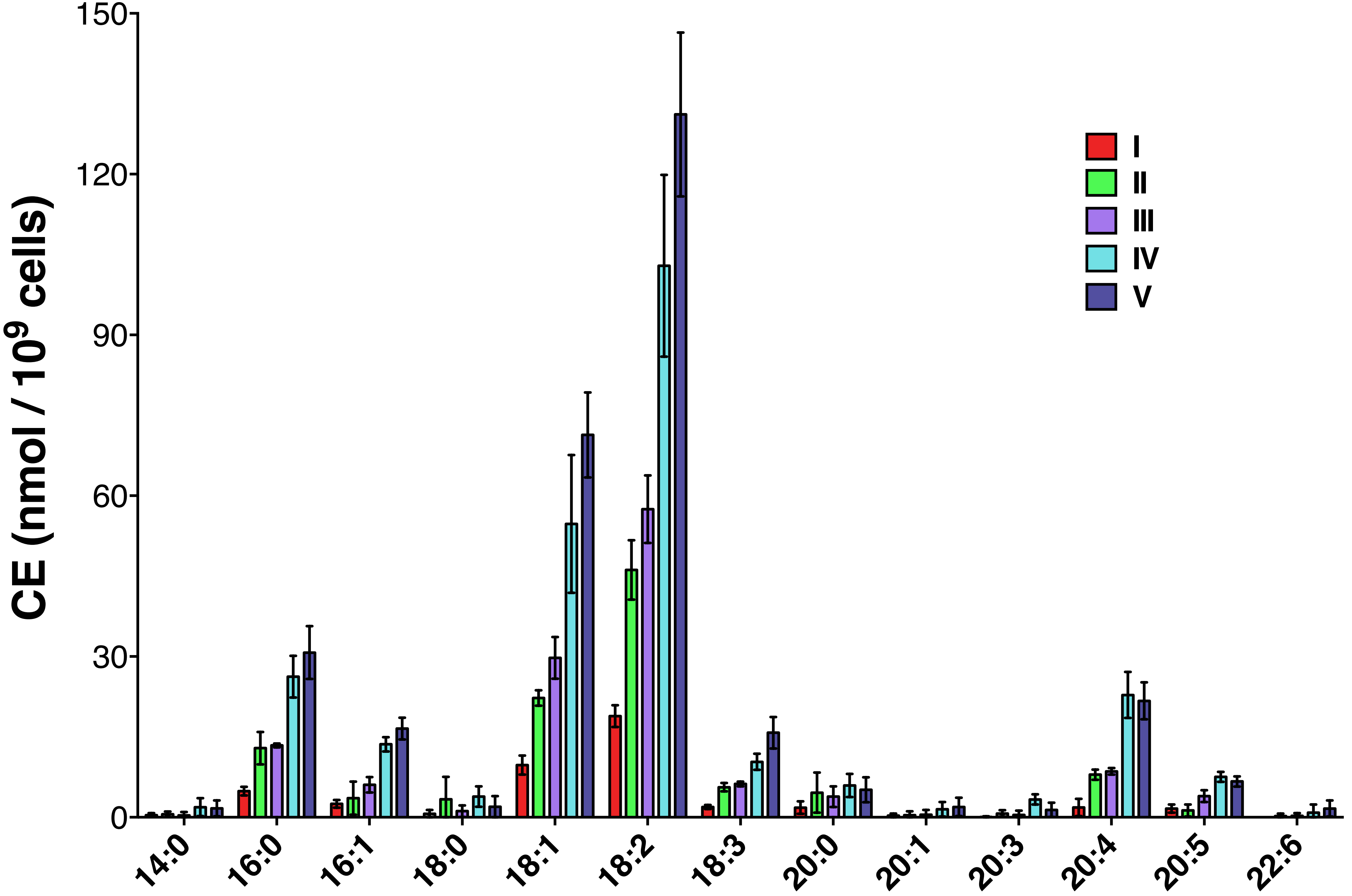

Additional file 5H.

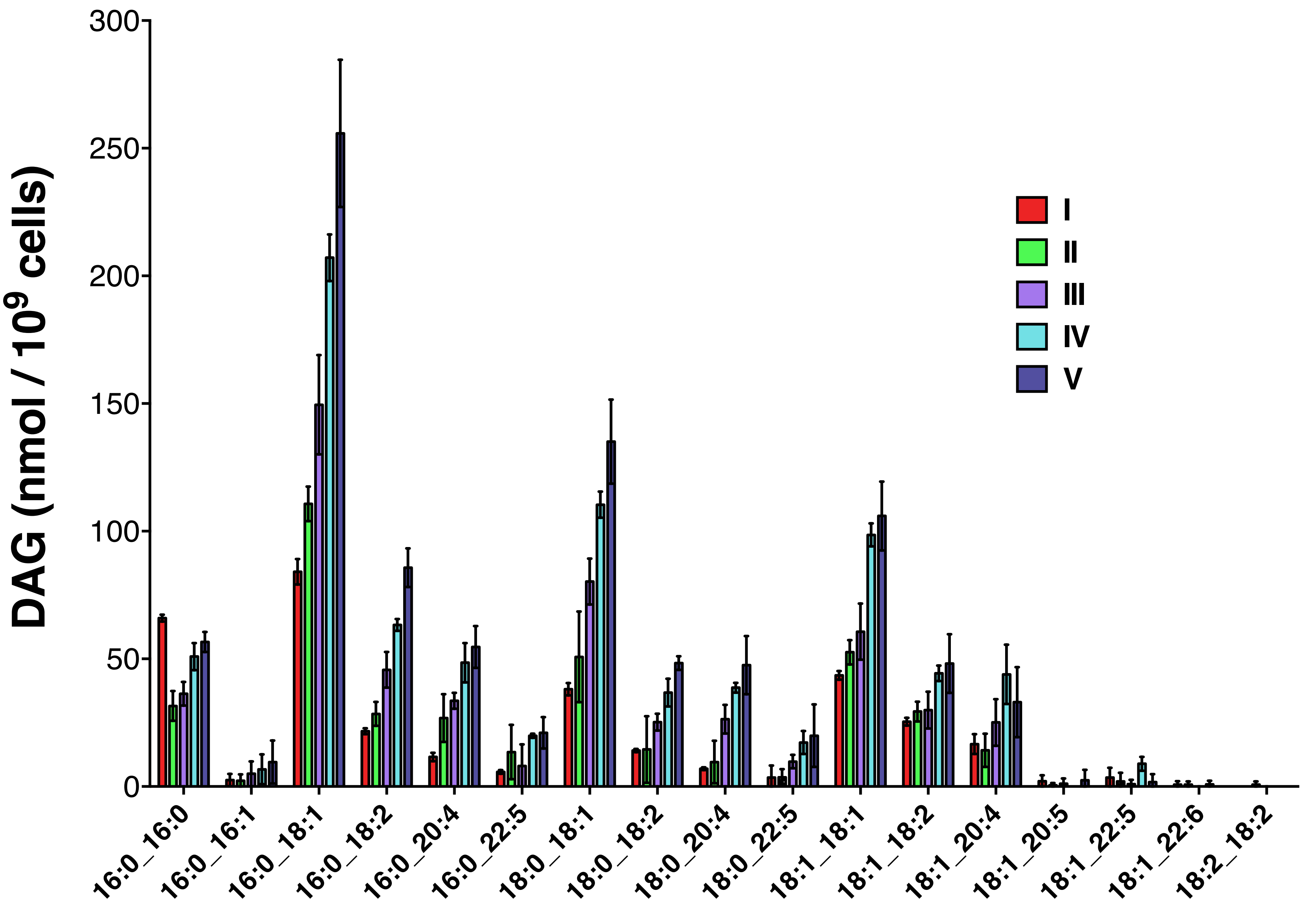

Additional file 5I.

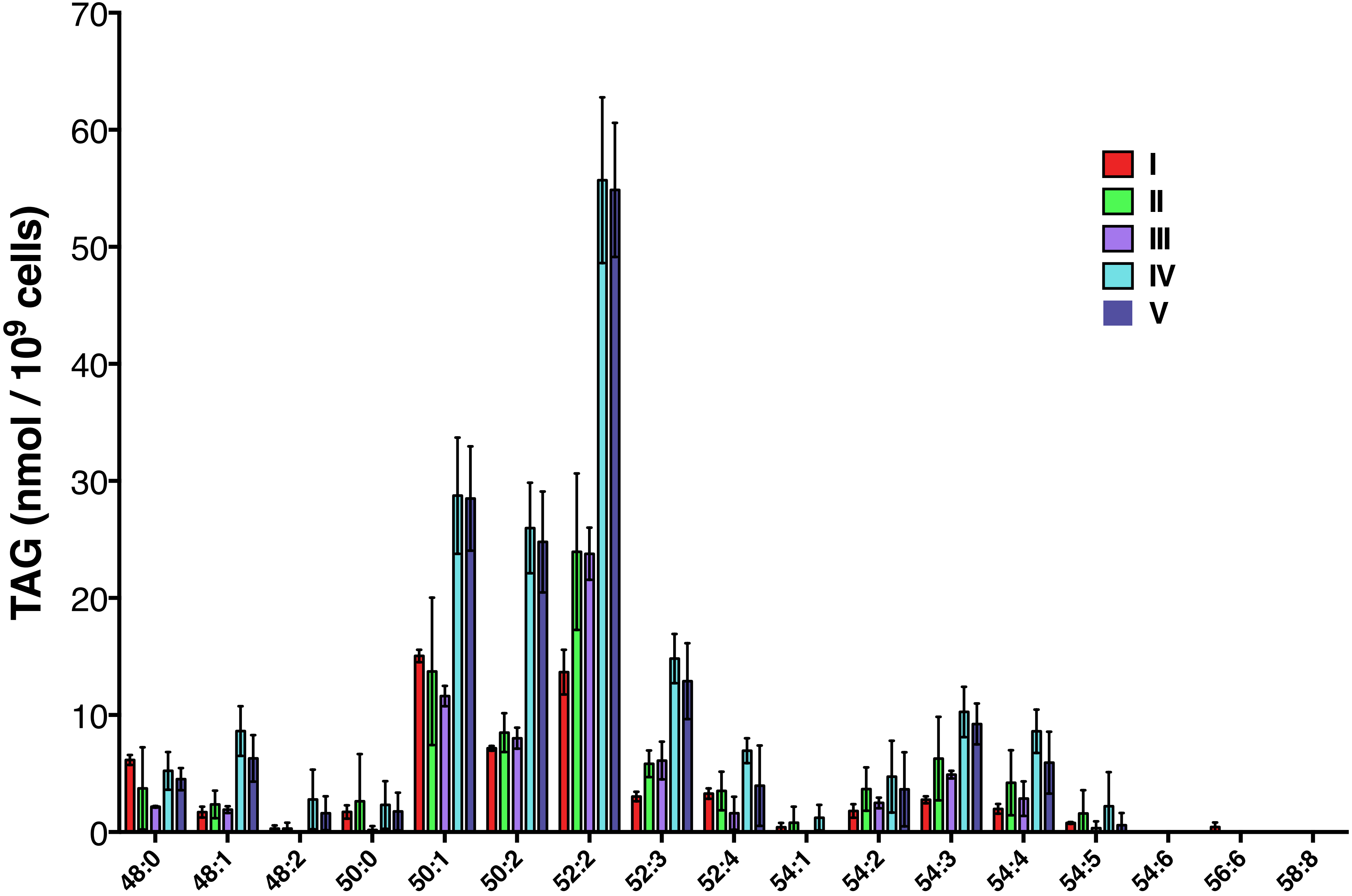

Supplement: Supplementary file 5 — 10.1186/s12936-016-1130-zContent of molecular lipid species of the blood-stage P. falciparum infected red blood cells. A. Sphingomyelin (SM). B. Ceramide (Cer). C. Phosphatidylcholine (PC). D. Phosphatidylethanolamine (PE). E. Phosphatidylserine (PS). F. Phosphatidylglycerol (PG). G. Cholesteryl ester (CE). H. Diacylglycerol (DAG). I. Triacylglycerol (TAG). I-V, gametocytes stage I to V; O, ether-linked lipids. Means and standard deviations of at least three independent experiments are shown. [file 12936_2016_1130_MOESM5_ESM.pdf]
